# Development of stealth liposomal formulation of celecoxib: *In vitro* and *in vivo* evaluation

**DOI:** 10.1371/journal.pone.0264518

**Published:** 2022-04-26

**Authors:** M. Yasmin Begum, Riyaz Ali M. Osmani, Ali Alqahtani, Mohammed Ghazwani, Umme Hani, Hissana Ather, Akhtar Atiya, Mohamed Rahamathulla, Ayesha Siddiqua

**Affiliations:** 1 Department of Pharmaceutics, College of Pharmacy, King Khalid University, Abha, Saudi Arabia; 2 Department of Pharmaceutics, JSS College of Pharmacy, JSS Academy of Higher Education & Research, Mysuru, Karnataka, India; 3 Department of Pharmacology, College of Pharmacy, King Khalid University, Abha, Saudi Arabia; 4 Department of Pharmaceutical Chemistry, College of Pharmacy, King Khalid University, Abha, Saudi Arabia; 5 Department of Pharmacognosy, College of Pharmacy, King Khalid University, Abha, Saudi Arabia; 6 Department of Clinical Pharmacy, College of Pharmacy, King Khalid University, Abha, Saudi Arabia; Bahauddin Zakariya University, PAKISTAN

## Abstract

Celecoxib (CLB) is a highly hydrophobic selective *cyclo-oxygenase* inhibitor with high plasma protein binding and undergoes extensive hepatic metabolism. CLB is highly effective in the treatment of osteo and rheumatoid arthritis as first line therapy but produces severe gastro-intestinal toxicities and cardiovascular side effects. In this research, stealth liposomes of CLB were developed with the intention to reduce the side effects and increase the accumulation of drug in the sites of inflammation. Stealth liposomes were prepared by thin film hydration technique using distearoylphosphatidylcholine and PE-PEG 2000 with variable amounts of cholesterol and characterized. The effects of various lipids such as hydrogenated soy phosphatidylcholine, dipalmitoyl phosphatidylcholine, distearoylphosphatidylcholine and cholesterol content on % drug encapsulation was investigated. The optimized stealth liposomes were characterized by FT-IR and DSC for possible drug excipients interaction. Pharmacokinetics, pharmacodynamics and biodistribution studies were carried out for the stealth liposomes. The results revealed that the stealth liposomes reduced the inflammation to the larger magnitude and have also sustained the magnitude when compared to free drug along with maximum analgesic response. Higher elimination half-life, AUC, MRT and lowered clearance rate denotes the extended bioavailability of the drug in blood. Biodistribution studies revealed that stealth liposomes extend the circulation time of liposomes in blood by decreasing opsonisation and be less concentrated in kidney, thereby reducing the toxicities to RES and renal organs and facilitate the drug accumulation in the area of inflammation. Our results indicated that CLB, without the requirement of modifications to enhance solubilisation, can be encapsulated and released from liposomal formulations. This new-fangled drug delivery approach may be used to circumvent the low bioavailability and toxic side effects of oral CLB formulations.

## 1. Introduction

Celecoxib (CLB) is an FDA-recommended non-steroidal anti-inflammatory drug (NSAID); which is implied as a first-line analgesic for patients experiencing severe pain as in osteoarthritis and rheumatoid arthritis. Designated chemically as 4-[5-(4-methylphenyl)-3-(trifluoromethyl)-1H-pyrazol-1-yl] benzenesulfonamide, CLB is a diaryl-substituted pyrazole. CLB is a highly selective *cyclo-oxygenase* (COX) inhibitor and it inhibits primarily the cyclo-oxygenase-2 (COX-2) isoform. It acts by selectively inhibiting COX-2; which helps in prostaglandin synthesis, an essential part of the inflammation and pain pathway. The inhibition of isoform COX-2 helps in reducing the number of inflammation mediators [1]. CLB is highly effective in the treatment of osteo and rheumatoid arthritis [2, 3] when compared to other NSAIDs such as naproxen and diclofenac. It is also used in the treatment of ankylosing spondylitis, colonic polyps and menstrual cramps. There are no liquid dosage forms that exists for CLB. The only oral dosage form available is capsule [4]. It was found that absorption of CLB is delayed by food [5]. CLB has poor flow characteristics and compressibility [6]. It is highly hydrophobic, absorbed completely after oral administration and undergoes extensive hepatic metabolism during its first passage through portal circulation [7]. CLB exhibits serious side effects such as gastrointestinal toxicities, gastric mucosal ulceration and hemorrhage due to inhibition of prostaglandin synthesis on oral administration that restricts oral use [8]. CLB has a wide volume of distribution of 455±166L, indicating its penetration into a number of tissues and organs [9, 10]. In addition, due to high plasma protein binding reported, i.e. 97% [11], it is recommended to be administered at high daily doses, thereby increasing the risk of cardiovascular side effects [12].

In general, it is well proven that the adverse effects of the drugs can be evaded by means of topical administration. This concept proved to be successful in the treatment of skin inflammatory disorders and/or when the target organ is easily accessible which is often not the case. In arthritis, intra articular administration can be the best way of realizing the local treatment. However, it needs a special technique to administer and causes osteo-necrosis due to the local reaction that takes place. To avoid this, a particular joint should not be injected more than 4 or 5 times per year. In addition, this treatment approach is only efficient when a single large joint is affected. But most cases of arthritis involve a number of joints affected by the disease and to treat this systemic therapy could be the right option. This lead to an insight into the development of CLB loaded novel delivery approach alternating the oral route of administration. Novel drug delivery carriers such as emulsions, microemulsions, micelles, microspheres and nanospheres have all been investigated but by far the most widely studied approach makes use of liposomes [13]. In addition, it was an evidence-based breakthrough in liposome research that the liposomes have the ability to localize in pathological sites spontaneously after intravenous (*i*.*v*.) administration by passive targeting. Thus, it was decided to prepare CLB incorporated liposome for *i*.*v*. therapy.

Though liposomes have valuable applications, their rapid and eminent elimination from the circulation by liver and spleen macrophages has seriously compromised their applications in conventional liposome formulations which contain saturated phospholipids and cholesterol. Specially to quote, after *i*.*v*. administration of conventional liposomes, they undergo binding with serum proteins such as fibronectin, immunoglobulin, C-reactive protein, etc., referred to as opsonins [14]. The process of opsonization is called as attaching of foreign particles to induce clearance by phagocytosis. Mononuclear Phagocyte System (MPS) present in organs such as the liver and spleen recognize these opsonins attached to the surface of liposomes and promote the clearance of liposomes from the blood circulation [14].

This drawback is associated with conventional liposomes, i.e., opsonization after *i*.*v*. administration can be eliminated by employing new second-generation liposomes called as stealth (or) sterically stabilized liposomes. Thus, CLB was preferred for this research assuming that loading CLB into stealth liposomes might reduce the side effects by increasing the accumulation of drug in the area of inflammation by passive targeting [15] and by reducing the availability of the drug in systemic circulation [16, 17].

## 2. Materials and methods

### 2.1. Materials

CLB was gifted by Aurobindo Pharma Pvt. Ltd, Hyderabad, India. Hydrogenated soy phosphatidylcholine (HSPC), distearoyl phosphatidyl choline (DSPC), PE 18:0/18:0-PEG_2000_ (PE-PEG) were generously donated by Lipoid, Germany. High purity cholesterol (CH) was procured from Sigma-Aldrich, St. Louis, MO, USA. All the other solvents and chemicals used in the present study were supplied by Himedia Laboratories Ltd., Mumbai, India and S.D. Fine Chemicals Pvt. Ltd., Mumbai, India, and were of analytical grade. Deionized water was used for all the studies.

### 2.2. Preparation of CLB loaded liposomes

The classic thin film hydration technique was adopted for the preparation of liposomes [17]. Specified quantities of CLB, lipids such as HSPC/DPPC/DSPC with or without cholesterol were taken in a 250 mL round bottom flask containing solvent system of chloroform and methanol (2:1, v/v). The solvent mixture was removed from the lipid phase at 45±2°C for HSPC and DPPC containing liposomes, and at 55±2°C for DSPC containing liposomes *via* rotary evaporation to obtain a thin film of lipids on the wall of the flask. Subsequently the flask was kept overnight under vacuum to ensure the complete removal of residual solvents. The dry lipid film was hydrated next day using phosphate buffer saline (PBS) pH 7.4. The resultant dispersion was vortexed for about 2 min. The dispersion was allowed to stand at rest for 2–3 h at room temperature to attain complete swelling of the lipid film so as to obtain a stable vesicular suspension. The obtained suspension was sonicated for sufficient period of time in an ultrasonic homogenizer (SFX550, Branson Ultrasonics, Emerson Electric Co., USA) and extruded through polycarbonate membrane of 0.2 μm pore size for 2 consecutive cycles [18, 19].

In all, fourteen varied batches of CLB loaded liposomes including conventional liposomes using HSPC, liposomes with long alkyl chain lipids such as DPPC, DSPC, and stealth liposomes using PE 18:0/18:0-PEG 2000 were prepared using different drug/lipid ratio and with variable amount of cholesterol by thin film hydration technique (**Table 1**).

**Table 1:** Formulae for various CLB loaded liposome formulations.

Sl. No.	CLB^#^ (mg)	HSPC^#^ (mg)	CH^#^ (mg)	DPPC^#^ (mg)	DSPC^#^ (mg)	PE-PEG^#^ (mg)	EE^#^ (%)*	Cumulative drug release over 24 h (%)*	Size (μm)*	Zeta potential (mV)
CL1	5	100	--	--	--	--	60.07±0.92	67.44±0.21	4.7±0.6	−12.91±2.3
CL2	7.5	100	--	--	--	--	68.22±0.76	68.23±0.08	4.6±0.2	−13.05±1.54
CL3	10	100	--	--	--	--	72.33±0.64	68.35±0.36	5.2±0.1	−13.57±1.73
CL4	15	100	--	--	--	--	52.44±0.65	65.39±0.33	4.5±0.3	−14.17±1.36
CL5	10	100	12	--	--	--	66.2±0.8	62.63±0.27	5.5±0.7	−12.39±1.22
CL6	10	100	24	--	--	--	59.23±0.84	53.73±0.24	4.8±0.4	−10.23±1.54
CL7	10	100	50	--	--	--	47.27±0.85	51.39±0.17	4.7±0.3	−9.5±1.83
CL8	10	--	--	100	--	--	91.47±1.02	47.77±0.98	5.9±0.2	−8.36±1.71
CL9	10	--	--	--	100	--	93.6±1.11	39.81±0.27	6.1±0.8	−9.47±2.16
CL10	10	--	12	--	100	--	90.7±1.21	35.49±0.38	6.2±0.3	−8.94±2.07
CL11	10	--	24	--	100	--	78.06±1.55	31.11±0.19	5.8±0.1	−7.67±1.19
CL12	10	--	50	--	100	--	65.9±1.31	22.81±0.23	5.7±0.8	−6.91±1.32
CL13	10	--	12	--	100	17	94.2±0.8	25.39±0.16	0.149± 0.25	−19.17±2.13
CL14	10	--	24	--	100	17	80.15±0.8	23.55±0.20	0.142± 0.8	−17.04±1.8
BCL13	--	--	12	--	100	17	--	--	0.137± 0.16	−17.63±1.49

#CLB-Celecoxib, HSPC-Hydrogenated soy phosphatidylcholine, CH-Cholesterol, DPPC-Dipalmitoyl phosphatidylcholine,

DSPC-Distearoylphosphatidylcholine, PE-PEG- PE 18:0/18:0-PEG_2000_, EE-Encapsulation efficiency, BCL13-Blank liposomes;

*Mean ± SD (*n* = 3)

### 2.3. Evaluation of CLB liposomes

#### 2.3.1. Separation of un-entrapped drug

Liposomes were sonicated and extruded through polycarbonate membrane of 0.2μm pore size to meet the required nano size of the vesicles. For these liposomes, the centrifugation was carried out to separate un-entrapped drug at 10000g at 4°C in refrigerated centrifuge for 2 cycles of 30 min with 10 min interval. The liposomal pellet was washed with 10 mL of PBS pH 7.4 for two times after decanting the supernatant and centrifuged again.

#### 2.3.2. Determination of percentage encapsulation efficiency (%EE)

10 mL of liposomal suspension was centrifuged at 10000g at 4°C for 30min and repeated twice with 10 min gap in between. Thus, the pellet obtained was collected and lysed in ethanol and sonicated for 10 min [19]. CLB concentration was determined by UV visible spectrophotometer at 251.2 nm. This was repeated thrice for each formulation and average was tabulated (Table 1). %EE was calculated using the following formula,

$$\%EE=\frac{Amount\;of\;drug\;in\;pellet}{Total\;drug\;amount}\times 100$$ (1)

#### 2.3.3. Fourier transform infrared (FT-IR) study

FT-IR study was carried out in in Magna IR 750 series II (Nicolet, USA) FTIR instrument by pelleting excipients of the stealth liposomes individually, pure drug and physical mixture of drug and excipients with IR grade KBr in the ratio of 1:100 at 15000 lb of pressure. The pellets were scanned in an inert atmosphere with the wave number range of 4000 cm^−1^ to 400 cm^−1^.

#### 2.3.4. Microscopy and vesicle distribution profile

A drop of liposome dispersion was placed on a glass slide, spread, examined for vesicles structure. The liposomal batches containing non-dispersed lipid film, aggregates or precipitates of the drug and insoluble drug residue were detected and discarded. The stealth liposomes size and its distribution were measured at 25±0.1°C in Zeta master apparatus (Malvern Instruments, Malvern, UK). Then the best stealth liposomes batch was observed with the help of scanning electron microscopy (SEM) for their morphology and topography [20, 21].

#### 2.3.5. Determination of zeta potential

Zeta potential was determined at 25°C by Zeta master apparatus (Malvern Instruments, Malvern, UK) taking 100 μl of sample and diluting it with 5 mL of water and injected in the electrophoretic cell at the potential of ±150 mV. Helmholtz—Smoluchosky equation in the instrument software was used to calculate the Zeta potential.

#### 2.3.6. Stability studies

The stability of the vesicles to retain the drug was assessed by storing the liposomal suspension at different temperature conditions such as freezer temperature (−20°C), refrigerator temperature (4-8°C), room temperature (25±2°C), 37±2°C and 45±2°C [13, 22, 23] for one month in sealed vials of 10 mL capacity. Samples were withdrawn periodically and drug content analysis was carried out as mentioned in the determination of %EE. Stability study was carried out over six months at accelerated and ambient conditions following ICH guidelines.

#### 2.3.7. Freeze drying (lyophilization)

The stealth liposomal suspension prepared along with cryoprotectant (lactose; 1:5 lipid–carbohydrate ratio) was rapidly frozen with iced acetone, overnight stored at −80°C and lyophilized for 48 h. Freeze dried product can be made in the form of suspension if needed for analysis in double distilled water within just less than 5 min of vortexing. Stability of freeze-dried product was tested for 6 months as per ICH guidelines and it is compared with suspension form of liposomes.

#### 2.3.8. Differential scanning calorimetric analysis

The analysis was conducted in differential scanning calorimeter (Model number TA– 60, Shimadzu, Japan) for all the constituents of the best stealth samples individually, CLB, CLB loaded and unloaded vesicles. In aluminium pan of differential scanning calorimeter 2 mg of sample was sealed hermetically and scanned at a scanning rate of 5°C/min between the temperature range of 25-225°C using nitrogen as the purge gas. For calibrating enthalpy indium was sealed in an aluminium pan with an empty pan as a reference.

#### 2.3.9. *In vitro* drug release

Modified dissolution apparatus (USP XXI dissolution rate model) that consists of a beaker (250 mL) and a plastic tube of diameter 17.5 mm opened from both the ends was used for studying in vitro drug release from liposomes [24, 25]. At one end of the tube sigma membrane with12000 MW cut-off was tied while the other end left free. This assembly was immersed into the beaker comprising 90 mL of dissolution medium at 37±1°C and beaker content was stirred at 100 rpm. Liposomal suspension of 10 mL was kept in the tube and sample of 1 mL was withdrawn periodically from the beaker every hour up to 24 h and analysed spectrophotometrically at 251.2 nm by UV spectrophotometer.

#### 2.3.10. Release kinetics

In order to find out the order and mode of drug release from liposome vesicles which was predominantly influence the drug release, the *in vitro* drug release data was subjected to diverse mathematical models and analysed. The numerous mathematical models and allied equations implied for the purpose are as follows:

For Zero order kinetic model:

$$Q_{t}=Q_{0}+K_{0}t$$ (2)

For First order kinetic model:

logQt=logQ0+K1t2.303 (3)

For Higuchi model:

Qt=KHt12 (4)

For Korsmeyer-Peppas model:

$$Q_{t}=at^{n}\;or\;f_{t}=at^{n}$$ (5)

Where Q_t_ is the amount of drug dissolved or drug released in time t; Q_0_ is initial amount of drug in the solution (most of the times Q_0_ = 0); K_0_ and K_1_ are the zero order and first order release constants, respectively; K_H_ is the Higuchi diffusion constant; n is the release exponent (indicative of the drug release mechanism) and function of t is M_t_/M_infinitive_ i.e. fractional release of drug [26].

### 2.4. *In vivo* evaluation

#### 2.4.1. Animals

Adult male albino rats were obtained from animal housing facility, Department of Pharmacology, Malla Reddy College of Pharmacy, Secunderabad (an approved and registered facility under CPCSEA 2010).

The experimental protocol for all *in vivo* studies was approved by Institutional Animal Ethical Committee, which is an approved body under CPCSEA 2010 (Registration number CPCSEA/MRCP/1217). Animals were maintained under controlled conditions of temperature and humidity in polypropylene cages filled with sterile paddy husk. They were fed with balanced diet [obtained from Mahaveera Enterprises (Registration number 146/1999/CPCSEA)] and water *ad libitum*.

#### 2.4.2. Anti-inflammatory activity of CLB liposomes

Anti-inflammatory activity of CLB loaded liposomes was tested by carrageenan induced rat paw edema method (CPCSEA/MRCP/1217).

Thirty Wistar strain adult male albino rats weighing 200±5 g were divided into 5 groups. Animals were marked on right hind paw of rats just behind the tibia-tarsal junction to confirm constant volume of paw when immersed into plethysmograph. Initial paw volume of the rats was measured just before carrageenan administration by dipping into plethysmograph. The sub plantar region of right hind paw of rats were injected with 0.1 mL of 1% w/v of carrageenan prepared in 0.9% normal saline. Group A was treated with 0.9% normal saline (Control group or ST-Saline treated group). Group B was treated with CLB solution (CS) through tail vein at the dose of 1 mg/kg body weight. Group C (CL), group D (DSPCL) and group E (SL) were treated with CLB conventional liposomes, liposomes made up of long alkyl chain lipid, DSPC and stealth liposomes intravenously at the dose of 1 mg/kg body weight [27]. Paw volumes of rats were measured for 8 h (at every 15 min for 1 h followed by every hour thereafter). The edema rate and %edema inhibition rate of each group was calculated using formulas [28] as follows:

$$Edema\;rate\;\left(E\%\right)=\left[\frac{\left(V_{t}-V_{0}\right)}{V_{0}}\right]\times 100$$ (6)

$$Inhibition\;rate\;\left(I\%\right)=\left[\frac{\left(E_{c}-E_{t}\right)}{E_{c}}\right]\times 100$$ (7)

Where, V_0_- Volume of paw just before injecting carrageenan (mL); Vt—Volume of paw after injecting carrageenan (mL); Ec—Control group’s edema rate; and Et—Treated group’s edema rate.

#### 2.4.3. Assessment of analgesic activity

*2*.*4*.*3*.*1*. *Tail flick test*. To confirm the analgesic effect of liposomes tail flick test [29] was conducted using a tail flick meter on mice. They were divided into 5 groups, with 6 animals in each group. Each group was named and treated as discussed in the above-mentioned section and injected intraperitoneally. The test was conducted by directing radiant heat on the tail’s dorsal surface. The time taken for the mice to withdraw tail against a noxious thermal stimulus was noted. The latency of 30 sec as maximum was constantly imposed on all animals to minimalize the tissue damage. The test was carried out every 15 minutes in the first hour followed by every one hour till 8 hours and then at 24^th^ hour.

*2*.*4*.*3*.*2 Hot plate test*. Hot plate test was conducted to confirm further the analgesic effect of stealth liposomes. Thirty mice were made into 5 groups, named and treated intraperitoneally as discussed in the above section. The mice were kept on aluminium hot plate at a temperature of 62±0.5°C for a maximum time of 30 sec [30]. The time taken for the mice to lick their fore and hind paws or jump was noted at 0, 0.25, 0.5, 0.75, 1, 2, 3, 4, 5, 6, 7, 8 and 24 h time period.

#### 2.4.4. Pharmacokinetics and bio distribution study

Male Wistar rats weighing about 200±10 g were used for the studies. 0.25 mL of Freund’s complete adjuvant [31] was injected intradermally into sub plantar region of right hind paw of rats. Development of arthritis was confirmed by difference in paw volume. After 3 weeks of injection, it was found that the difference in thickness of the right and left paw was 6.83±0.26 mm.

Arthritic animals were made into 4 groups with 15 animals in each group. Group A was injected with 1 mg/kg CLB solution via tail vein intravenously (CS) and group B with HSPC liposomes (CL), group C with liposomes containing DSPC (DSPCL) and group D with stealth liposomes (SL). Blood samples were collected by retro orbit puncture in heparinized capillaries at stated time intervals and then kept in heparinized centrifuge tubes. Pharmacokinetic parameters such as elimination half-life, area under plasma concentration time curve (AUC), mean residence time (MRT) and clearance were calculated using NCOMP-A Windows based computer program for non-compartmental analysis.

Tissues such as liver, spleen, kidney and paw were dissected after sacrificing the rats for conducting bio-distribution studies, weighed and stored at −20°C until further analysis. Each organ was homogenized in methanol keeping in ice bath, centrifuged at 4500 rpm. CLB content was analysed in supernatant by high performance liquid chromatography (HPLC).

#### 2.4.5. Analysis CLB in methanol by HPLC

*Instrumentation*. Waters Alliance HPLC system (Milford, USA) with 2695 separations module with auto sampler and column oven was used. The configurations of the system were 501 solvent delivery system (Pump), Rheodyne 7125 injector with variable capacity loop and 2487 dual λ absorbance detector with Empower 2 operating software and with system suitability.

*Chromatographic conditions*. Kromasil C18 (5 μm, 250 mm X 4.6 mm i.d) column was used as stationary phase and acetonitrile: phosphate buffer pH 7, in the ratio of 65:35 v/v as mobile phase. The mobile phase was filtered through 0.45 μ membrane filter and degassed before analysis. The flow rate was 0.8 mL/min and the column effluent was monitored at 254 nm.

*Preparation of standard solution of CLB*. A 10 mg/mL solution was prepared in methanol. Serial dilutions were made to get the resultant concentrations of 200 μg/mL to 1000 μg/mL.

*Calibration curve of CLB in plasma*. Blank plasma of 60 μL was spiked with 40 μL standard solutions of CLB (10 μg/mL) in a micro centrifuge and 1 mL of methanol was added. The tubes were vortexed and then centrifuged at 4000 rpm for 5 min, after centrifugation, an aliquot 20 μL supernatant solution was injected to HPLC. Procedure was repeated to prepare resultant concentrations of 200 μg/mL to 1000 μg/mL.

*Estimation of CLB from the plasma samples*. A plasma sample of 100 μL was taken in a micro centrifuge tubes and 1 mL of methanol was added. The tubes were vortexed, then centrifuged at 4000 rpm for 5 min. After centrifugation, an aliquot 20 μL supernatant solution was injected to HPLC. Same procedure was repeated for 1, 2, 4, 8, and 24 h. Pharmacokinetic data was made by NCOMP- A Windows based computer program.

#### 2.4.6. Statistical analysis

All the recorded research data; wherever mentioned, was statistically analysed using GraphPad Instat. 8.0.2 software (La Jolla, CA). Results are mostly expressed as mean ± SD for (*n* = 3 to 15) independent experiments. One-way ANOVA with post-hoc analysis implying Dunnett’s Multiple Comparisons Test was used to assess differences amongst the groups. *p* < 0.05 was considered as statistically significant difference amid all the groups [a: *p* < 0.05, b: *p* < 0.01, c: *p* < 0.001, and d: *p* < 0.0001 (in comparison to the control group)].

## 3. Results and discussion

### 3.1. Formulation development and *in vitro* evaluation of CLB liposomes

#### 3.1.1. Optimization of process parameters

As a preliminary study, the formulation technique was optimized by studying various process parameters such as speed of rotation of rotary evaporator, vacuum pressure, medium of hydration and time of hydration. The optimized process parameters for the preparation of CLB liposomes were as follows:

Speed of rotation– 60 rpm for conventional liposomes prepared using HSPC; 150 rpm for liposomes with long alkyl chain lipids such as DPPC, DSPC; 120 rpm for stealth liposomes.
Vacuum pressure– 250 mmHg
Temperature–Evaporation temperature: 45±2°C for HSPC and DPPC containing liposomes; 55±2°C for DSPC liposomes.
Hydration temperature: 60±2°C for all the liposomes.
Hydration medium–PBS (pH 7.4)
Hydration time– 2 min by vortexing.
Time of sonication– 3 min for conventional liposomes and liposomes with long alkyl chain lipids; 5 min for stealth liposomes.
Chloroform: methanol (2:1, v/v) was used as the solvents system because this has a lower contact angle with glass than other solvents and thus chloroform: methanol (2:1, v/v) has fewer tendencies to give thick deposits of lipids at all edges while drying down.

#### 3.1.2. Preparation of CLB loaded liposomes

Fourteen liposome formulations (CL1 to CL14) were prepared using varying drug-lipid ratio, phospholipids and with or without cholesterol by thin film hydration technique (**Table 1**).

*3*.*1*.*2*.*1*. *Effect of drug-lipid ratio on %EE of liposomes*. First four liposome formulations (CL1 to CL4) were prepared using only HSPC without cholesterol to find out the influence of drug-lipid ratio on %EE of the liposomes. It was found that drug-lipid ratio used in the preparation of the vesicles plays the important role in development of liposomes because %EE of liposomes was found to be dependent on the drug-lipid ratio. CLB encapsulation into liposomes was found to increase while attempting to increase amount of CLB from 5 mg (Drug: HSPC, 1:10 molar ratio) to 10 mg (Drug: HSPC, 1:5 molar ratio) and %EE increases considerably from 60.07±0.92% to 72.33±0.64% (Table 1). However, %EE started decreasing with further increase in quantity of the drug used. In all the four formulations the amount of the lipid used was fixed i.e. 100 mg and drug—lipid ratio of 1:10% w/w showed the greater drug entrapment (**Table 1**).

*3*.*1*.*2*.*2*. *The influence of alkyl chain length of lipids on %EE of CLB liposomes*. Two batches of liposomes (CL8 and CL9) were prepared containing 2 different lipids with varying alkyl chain length such as DPPC (16 alkyl chain length) and DSPC (18 alkyl chain length) which were compared with HSPC (natural phospholipids containing 3 alkyl chain length). These two formulations were prepared without cholesterol using 10 mg of drug and 100 mg of DPPC/DSPC (Drug-lipid molar ratio of 1:5) respectively. Our results indicate that lipids with increasing alkyl chain length increases %EE in the following order, DSPC>DPPC>HSPC (**Table 1**). This might be due to the fact that incorporation of longer alkyl chain lipids increases the hydrophobic area in the bilayer lipid membrane.

*3*.*1*.*2*.*3*. *Influence of cholesterol on %EE of CLB conventional liposomes and liposomes prepared with long alkyl chain lipids*. In order to find out the influence of cholesterol on CLB encapsulation into liposomes, three formulations (CL5, CL6 and CL7) were prepared. It was found that there exists influence of cholesterol on %EE of liposomes. Liposomes prepared with HSPC and zero cholesterol were found to have the highest %EE i.e. 72.33±0.64, whereas liposomes prepared incorporating cholesterol have shown the %EE in the following order CL5 >CL6 >CL7 [HSPC/Cholesterol molar ratio of 4:1 (CL5), 2:1 (CL6), 1:1 (CL7) (**Table 1**)]. This result might be due to the competing nature of drug and cholesterol for getting accommodated into bilayer membrane. It is well known that cholesterol provides rigidity to the bilayer membrane. It reduces the permeability and increases the retention of the solute. Thus, incorporation of cholesterol is very much essential for the preparation of stable liposomes. The results obtained with conventional liposomes were further confirmed with the study of influence of cholesterol on %EE of liposomes prepared with long alkyl chain lipids. Liposomes prepared with DSPC and zero cholesterol was found to have greater %EE than the other liposomes prepared incorporating cholesterol. Three formulations were prepared using different DSPC/cholesterol molar ratio of 4:1, 2:1 and 1:1. Along with 100 mg of DSPC, 12 mg, 24 mg and 50 mg of the cholesterol respectively was used in these three formulations. It was noted that presence of cholesterol reduces %EE in the following order CL9>CL10>CL11>CL12 interestingly, although DSPC/cholesterol composition of CL12 routinely used as starting composition regarding conventional liposomes. Later when we thoroughly gone through the literature, we came to know that high content of cholesterol may decrease the %EE of hydrophobic molecules. Some of the earlier reported findings are: liposomal MLVs prepared with egg phosphatidyl choline could encapsulate 29.5% of ibuprofen, whereas ibuprofen encapsulation gets reduced to 23.2% with 30% of cholesterol and even to 17.1% with 50% of cholesterol [16]. In a study of developing cremophor-EL free liposomal paclitaxel formulation, it was experiential that increasing the content of cholesterol had decreased the drug loading efficiency dramatically from 99.3% to 6.2% [32]. However, in some cases increasing amount of cholesterol have showed increased drug encapsulation efficiency, for example Bhatia *et al*. have reported 30% cholesterol addition during formulation led to increased drug entrapment efficiency from 45.2% to 57.5% [33]. So, there are variable outcomes reported for the encapsulation efficiency of hydrophobic molecules due to the increasing or decreasing amount of cholesterol. These effects may be due to molecular interaction between the phospholipids, cholesterol and the drug. But in general, cholesterol increases the hydrophobicity of the bilayer membrane which may favour the inclusion of hydrophobic molecules [34, 35]. On the other hand, considering the conflicting fact that cholesterol may prefer to accommodate in the hydrophobic bilayer structure which is having a limited space. So, there might be the competition between cholesterol and drug in getting aligned themselves for this space between the alkyl chains of phospholipids that results in higher encapsulation when there is no cholesterol and lower encapsulation with increasing cholesterol content. Furthermore, it has also been reported that ionic strength of lipid mixture used in developing liposomes greatly influences mean diameter of resultant liposomes based on the content of charged lipids [33]. Thus, one can keep control on particle size by varying charged lipid components proportion.

The use of HSPC in formulation resulted in liposomes with smaller mean particle sizes and lower polydispersity indexes with respect to the DSPC based liposomes; which perfectly aligns with earlier reports in the literature [23, 34]. Whereas, in comparison to these the particle size of PE-PEGylated liposomes was noted to be even smaller, which confirms and signifies the superiority of PE-PEGylated formulations over conventional one both in terms extended circulation and permeation and is in agreement with the similar studies reported in literature [32, 35, 36]. This noted reduced particle size could be attributed to the curving of the bilayer for reducing the intensity of lateral repulsion; which is caused by the addition of increasing PE-PEG amounts in the lipid bilayer. The PE-PEGylated lipid also leads to interlamellar repulsion that leads to decrease in lamellarity and thus particle size; which is a well-established trend [33, 36].

*3*.*1*.*2*.*4*. *Preparation of CLB stealth liposomes and its effect on %EE*. The final phase of our study focused on preparation of long circulating liposomes commonly known as stealth liposomes using thin film hydration technique. As per our studies, the drug-lipid ratio, lipid cholesterol ratio and the best lipid which provide greater drug entrapment were chosen for the preparation of stealth liposomes. Polymer lipid PE 18:0/18:0-PEG 2000 (polymer of polyethylene glycol covalently attached to phosphatidyl ethanolamine) was used to provide steric stabilization. Increase in drug-lipid molar ratio above and below 1:5 was found to limit the drug encapsulation efficiency. Drug encapsulated stealth liposomes were prepared using the combination of DSPC: Cholesterol: PE-PEG in the molar ratio of 4:1:0.2 and DSPC: Cholesterol: PE-PEG in the molar ratio of 4:1:0.4. The former one was found to have greater drug entrapment than the liposomes prepared with only DSPC/cholesterol. In view of the observations obtained, further increasing the amount of PE-PEG to 2:1:0.2 (DSPC: CH: PE-PEG) molar ratio led to dramatic decrease in drug encapsulation and stability (data not shown in table).

#### 3.1.3. Fourier transform-infrared (FT-IR) study

Drug, excipients interaction was studied before developing the formulation by using FTIR-spectroscopy, which is one of the most important analyses to describe about the stability of formulation, presence of drug and its compatibility with used excipients.

**Fig 1:**
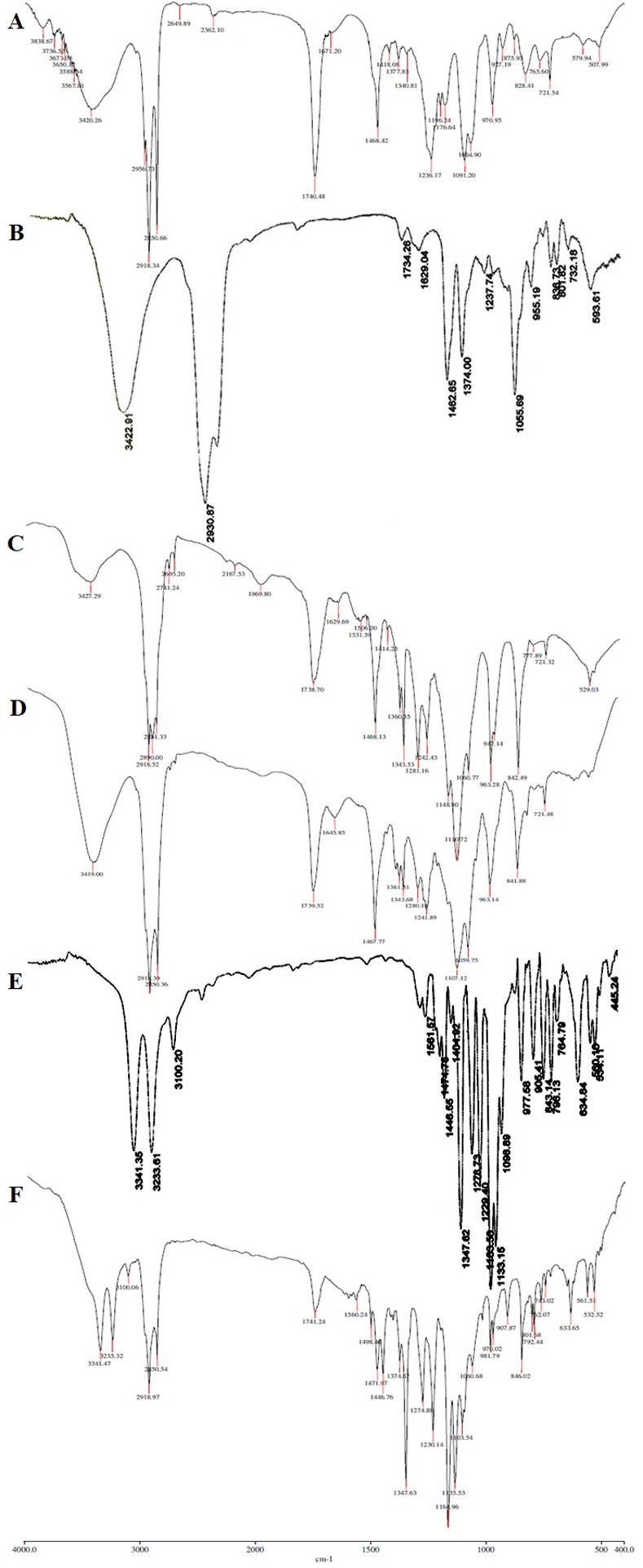
Overlain FT-IR spectra of (A) pure DSPC, (B) pure cholesterol, (C) pure PE-PEG, (D) physical mixture of excipients for the formulation, (E) pure CLB, and (F) physical mixture of CLB and excipients for the formulation CL13.

**Fig 1D** shows minor shifting of some peaks compared with individual excipients (**Fig 1A–1C**), like aliphatic N-H stretch (3420.26 to 3419.00 cm^−1^), C-H stretch (2918.52 to 2918.39 cm^−1^), C = O stretch of ester (1468.13 to 1467.77 cm^−1^), C-O stretch of ester (1374.00 to 1343.68 cm^−1^), C-O stretch of hydroxyl group (1060.77 to 1059.75 cm^−1^). Minor shifts were observed when spectrum in **Fig 1F** was compared with spectrum of pure drug (**Fig 1E**) and the individual excipients (Fig 1A–1C) such as aliphatic N-H stretch (3341.35 to 3341.47 cm^−1^), C-H stretch (2918.52 to 2918.97 cm^−1^), C = O stretch of ester (1468.13 to 1471.97 cm^−1^), C-O stretch of ester (1347.62 to 1347.63 cm^−1^), and C-O stretch of hydroxyl group (1060.77 to 1060.68 cm^−1^).

Over all, these minor shifts observed due to the formation of hydrogen bonds, Vander Waals attractive forces or dipole moment which are weak forces seen in the polar functional groups of drug and excipients. The frequency of absorption due to the carbonyl group depends mainly on the force constant which in turn depends upon inductive effect, conjugative effect, field effect, stearic effects. The minor shifts observed due to the interactions mentioned above might support producing required vesicle shape, good stability and drug release sustainability.

#### 3.1.4. Vesicle size and its distribution

**Fig 2:**
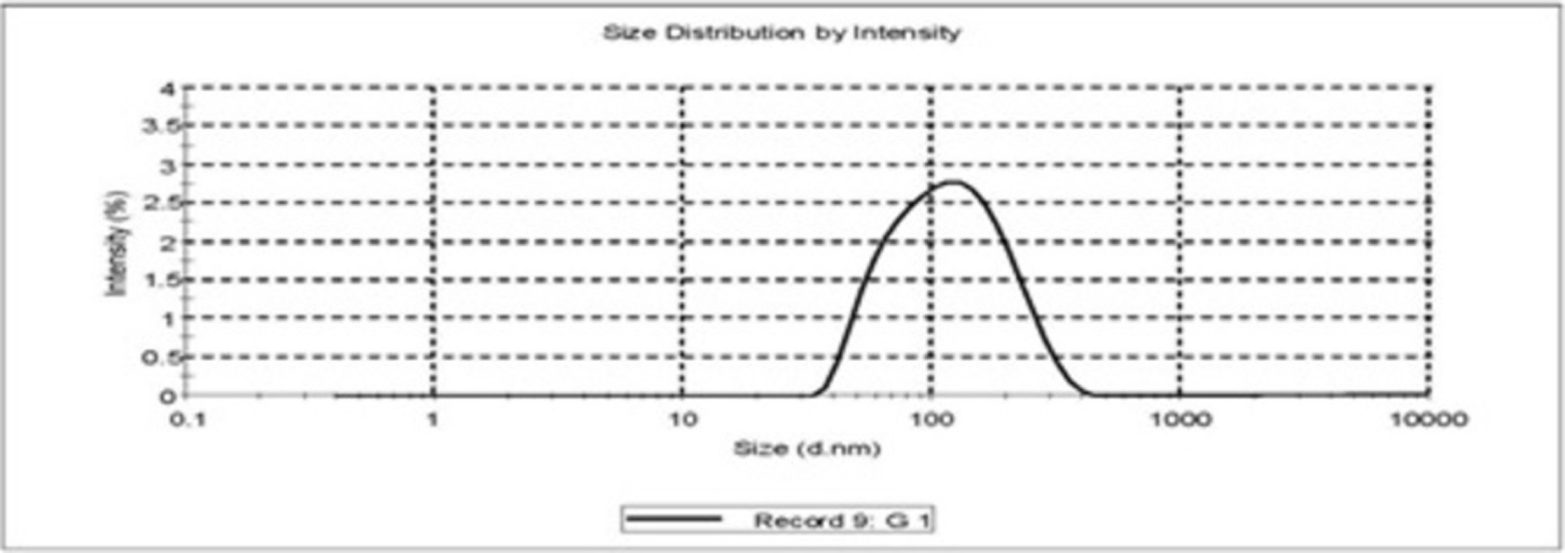
Vesicle size distribution of stealth liposomes (CL13).

**Fig 3:**
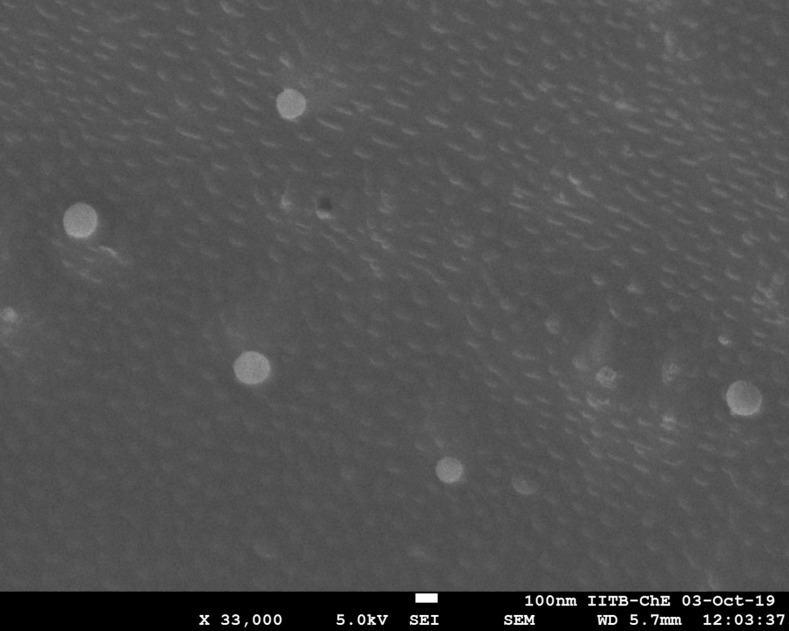
SEM image of stealth liposomes (CL13).

The vesicle size of the liposome formulations prepared without sonication and extrusion cycle was found to be in the range of 4.5±0.3 to 6.2±0.3 μm. Most of the vesicles were found to be spherical in shape. Size analysis was repeated for 3 formulations of each formulation code and vesicle size data was compared (**Table 1**). Data was found to be highly reproducible every time. The SEM photograph of the best optimized liposome formulation CL13 showed that the vesicles were homogeneous and spherical in shape and showed the vesicle size of 149±0.25 nm after subjecting the liposomal dispersion to sonication and extrusion cycles (**Figs 2 and 3**).

#### 3.1.5. Zeta potential measurements

**Fig 4:**
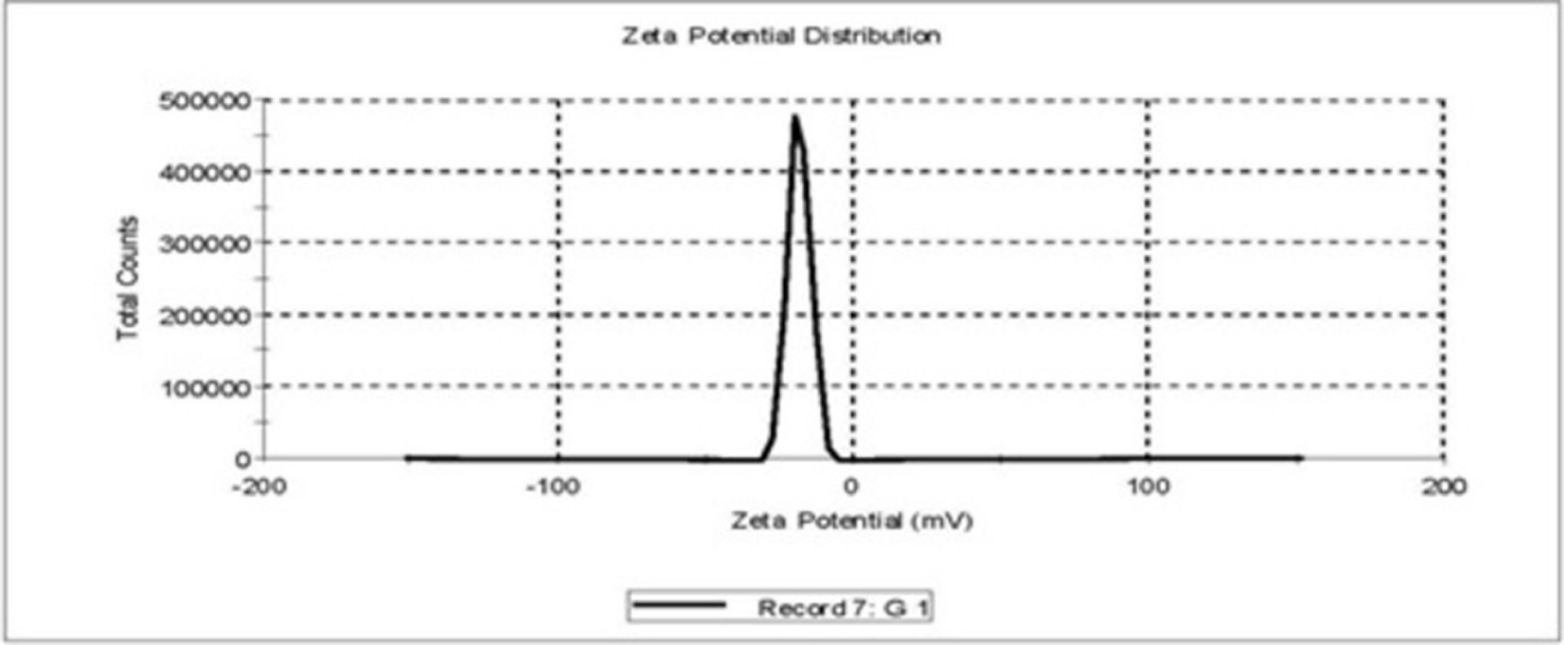
Zeta potential distribution graph of stealth liposomes (CL 13).

Zeta potential values noted for all formulations are presented in Table 1. Zeta potential for CL13 (stealth liposomes) liposomes were found to be −19.17±2.13 and −19.75±3.72 at 0 h and 24 h respectively, with good result quality. This showed that CL13 formulation was noted to be appreciably stable (**Fig 4**).

#### 3.1.6. Stability profile

**Fig 5:**
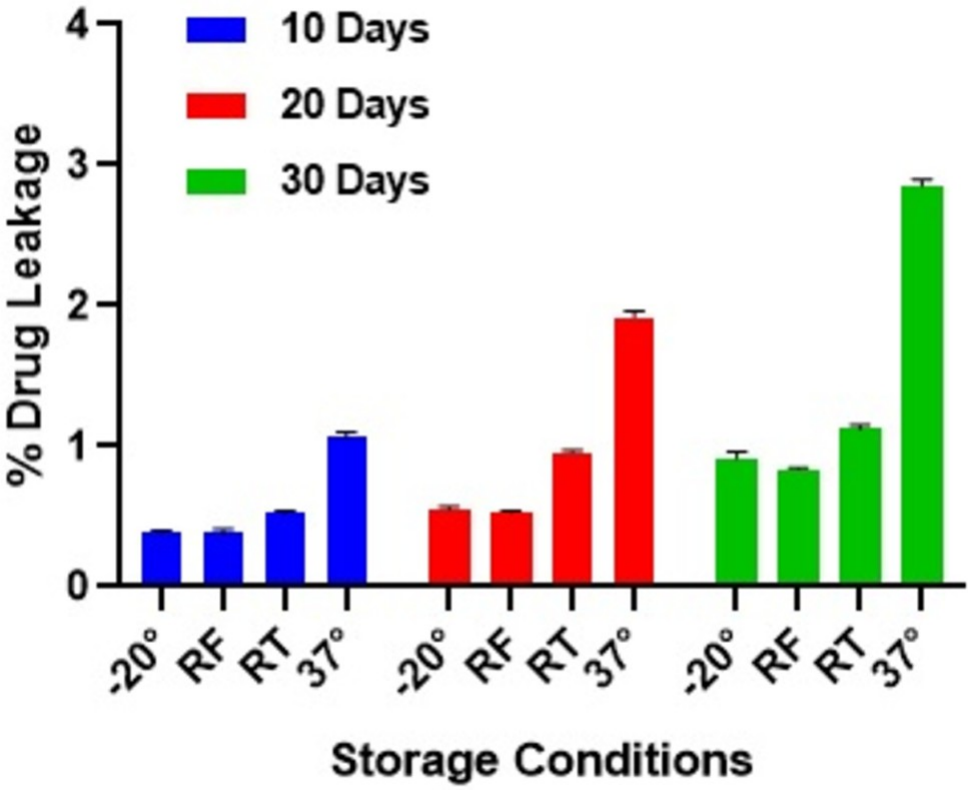
Extent of drug leakage from CL13 liposomes at different storage conditions.

To ensure the physical and chemical stability of liposomes, the best stealth liposomes was stored at 4 different temperatures such as −20°C, 4°C, 25°C and 37°C for 1 month. There were no significant changes in %EE for the formulations stored at −20°C and 4°C. The % drug leakage varied from 1–3% for the samples stored at 25°C as well as 37°C (**Fig 5**). The compiled 6 months stability data of accelerated and ambient conditions as per ICH guidelines is shown in **Table 2**. The 6 months accelerated stability data indicated that the formulation was stable as far as assay was concerned and assured that the process for preparing liposomes was reproducible and produced excellent results.

**Table 2:** Stability studies data of stealth liposome formulation CL13 as per ICH guidelines.

Test	Specification	Initial	25 ± 2°C/ 60 ± 5% RH				5 ± 3°C			
			Time (months)							
			1	2	3	6	1	2	3	6
Assay (%w/w)	95 to 105%	99.52±0.42	98.88±0.31	97.73±0.26	96.67±0.37	94.69±0.51	98.73±0.43	98.33±0.39	97.92±0.34	95.85±0.43

Data expressed as mean ± SD, *n* = 3

#### 3.1.7. Freeze drying (lyophilization)

The physical and chemical instability problems associated with liposomes such as hydrolysis, oxidation, leakage of the encapsulated drug and alterations in vesicle size due to fusion and aggregation could be reduced by freeze drying the liposomal suspension using suitable cryoprotectant. In our study lactose was used as a cryoprotectant. For freeze drying, liposomal suspension was prepared with cryoprotectant (lactose; 1:5 lipid-carbohydrate ratio). The freshly prepared liposomal suspension was enriched with lactose solution and quickly frozen with iced acetone, stored at −80°C overnight and lyophilized for 48 h using freeze dryer. Before measurements the lyophilized samples were re-suspended in double distilled water. Rehydration process is completed in 5 min by vortexing.

Stability study was conducted for 6 months at accelerated temperature (25±2°C/60±5% RH) and ambient temperature (5±3°C) for the freeze-dried product of CL13 (Stealth liposomes) and the data was compared with stability data of CL13 liposomal suspension. Comparative stability data of CL13 liposomal suspension and freeze-dried product is shown in **Table 3**.

**Table 3:** Comparative stability profile of liposomal suspension and freeze-dried form of CL13 formulation as per ICH guidelines.

Test	Specification	Sample Name	Initial	25 ± 2°C/ 60 ± 5% RH				5 ± 3°C			
				Time (months)							
				1	2	3	6	1	2	3	6
Assay (%w/w)	95 to 105%	CL13 suspension	99.52±0.42	98.88±0.31	97.73±0.26	96.67±0.37	94.69±0.51	98.73±0.43	98.33±0.39	97.92±0.34	95.85±0.43
		CL13 freeze-dried product	99.71±0.38	99.04±0.17	98.56±0.31	97.96±0.29	96.75±0.45	99.52±0.33	99.41±0.28	99.18±0.19	98.36±0.37

Data expressed as mean ± SD, *n* = 3

The 6 months accelerated stability data indicated that both the forms of products were stable as far as assay was concerned. Amongst them the freeze-dried product was found to retain more drug at each sampling point. Hence freeze-dried product possesses greater stability than the suspension form. At each sampling point, negligible changes in vesicle size were observed (**Table 4**) for freeze dried product when compared to liposomal suspension. The possible reason for good stability of the optimized formulation could be the optimized process as well as formulation factors.

**Table 4:** Comparative data for changes in vesicle size of CL13 at ambient storage condition over 6 months period.

Name of the sample	Vesicle size (μm) at 5 ± 3°C storage condition				
	Initial	1 month	2 months	3 months	6 months
CL13 suspension	0.149±0.25	0.156±0.39	0.163±0.41	0.171±0.27	0.194±0.58
CL13 freeze dried product	0.146±0.34	0.152±0.28	0.155±0.32	0.157±0.49	0.172±0.92

Data expressed as mean ± SD, *n* = 3

#### 3.1.8. Differential scanning calorimetry analysis

A single sharp peak was observed corresponding to the phase transition temperatures of drug and excipients such as at 54.9±0.1°C for DSPC, 150.5±0.1°C for cholesterol, 56.1±0.1°C for PE-PEG and at 163.24±0.1°C for CLB.

**Fig 6:**
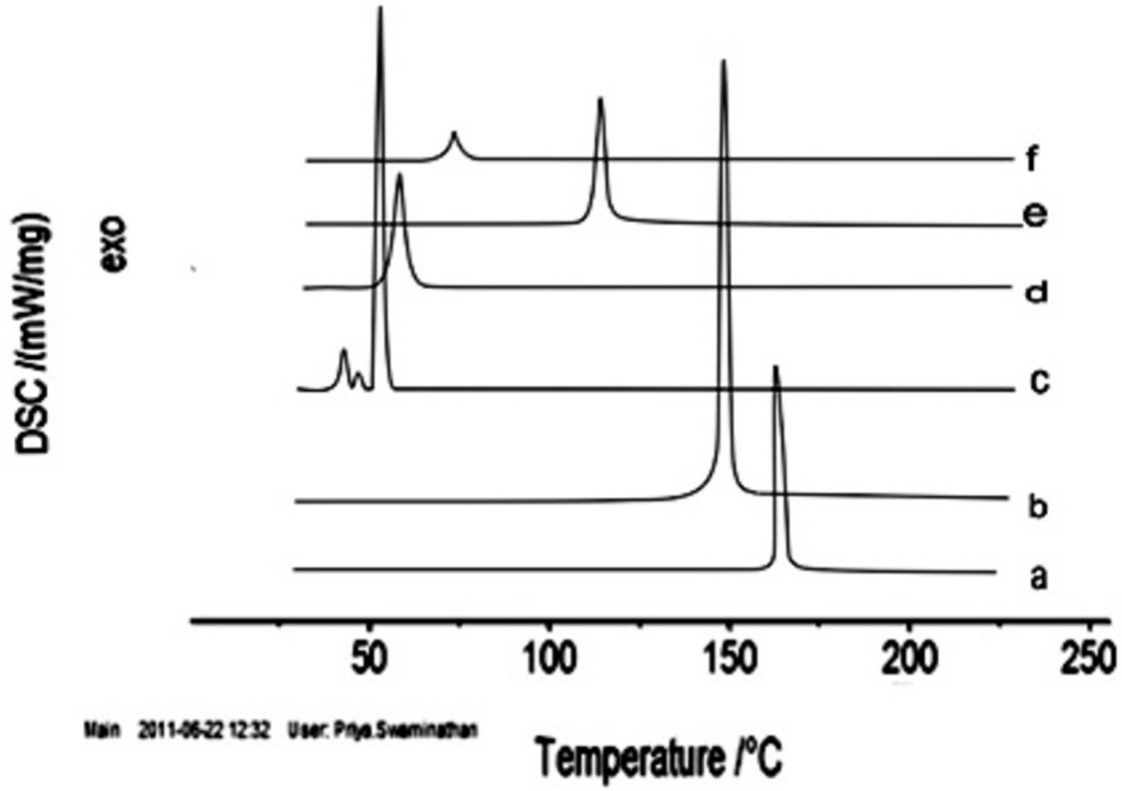
DSC thermograms of (a) pure drug CLB, (b) cholesterol, (c) DSPC, (d) PE-PEG, (e) CLB loaded liposomes, and (f) CLB unloaded liposomes.

Thermogram of CLB loaded liposomes (**Fig 6**) depicted an exothermic peak at 118.5±0.1°C and that in case of unloaded liposomes was observed at 71±0.1°C. Since all the above-mentioned DSC thermograms exhibited prominent exothermic peaks above 40°C, the results satisfy the prerequisite of maintaining liposomes in solid state at the body temperature. In case of CLB loaded liposomes, there was no CLB peak identified in the thermogram, and the peak of DSPC was found to be shifted from 54.9° to 118.5°. Not only DSPC but other components peak also might have shifted to 118.5°. These results signify that DSPC and cholesterol interacted with each other to a great extent while forming the lipid bilayer and there might be a significant interaction of CLB with phospholipid bilayers aiding to enhance drug entrapment and decreased the release rate.

#### 3.1.9. *In vitro* drug release

**Fig 7:**
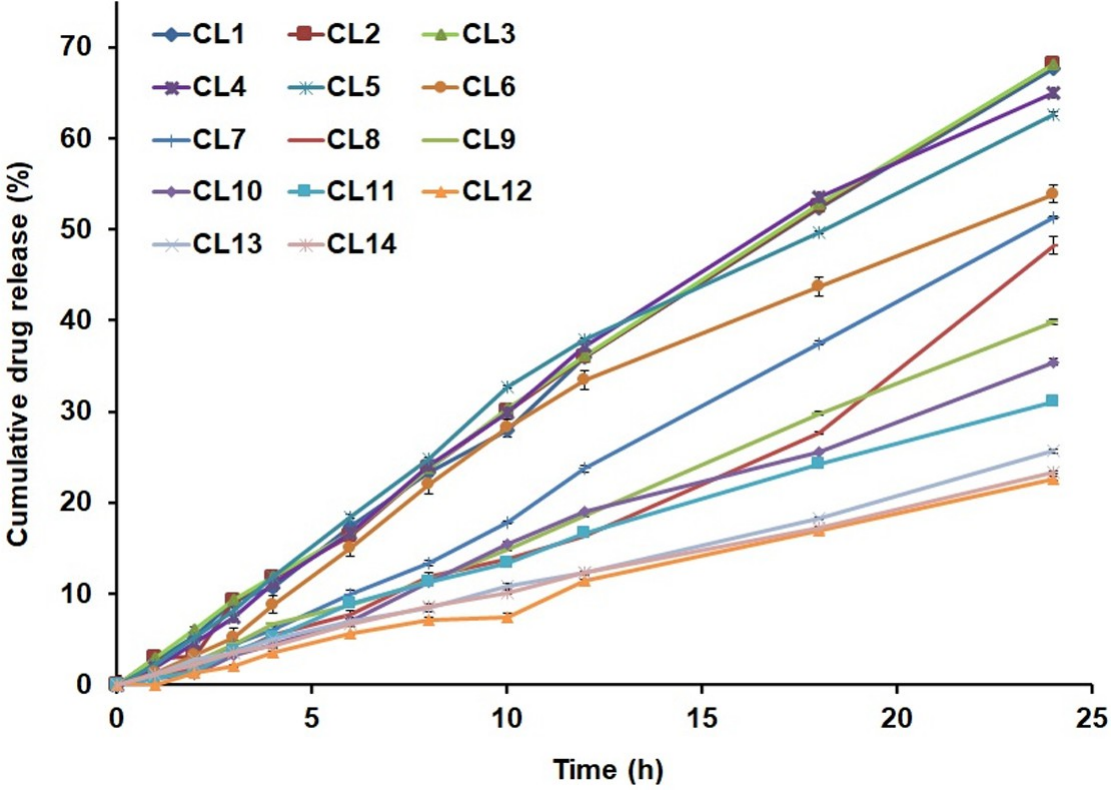
Comparative in-vitro drug release profiles of formulations CL1-CL14 over 24 h time period.

Among the various formulations prepared, CL3 could release the highest amount of CLB i.e., 68.35±0.33% in 24 h. CL3 contains 1:10% w/w of drug, HSPC (Drug/HSPC molar ratio of 1:5) and zero cholesterol (**Table 1**). Then comes in order the formulations CL5, CL6 and CL7 (**Fig 7**) comprising 4:1, 2:1 and 1:1 molar ratio of HSPC and cholesterol. The drug release percentages for these formulations were noted to be 62.63±0.27%, 53.73±0.24% and 51.39±0.08%, respectively. There was no burst release observed in any of the formulations. It was observed that cumulative release profile of CLB is much similar for liposomes containing low concentration of cholesterol also. But 4:1 molar ratio of HSPC and cholesterol (CL5, 100 mg of HSPC and 12 mg of cholesterol) might be the right choice for liposomal formulations of CLB since there is significant reduction in CLB encapsulation at high cholesterol concentrations.

Among the formulations prepared using long alkyl chain lipids such as DPPC and DSPC, CL8 liposomes prepared with DPPC alone could release the highest amount of the drug i.e., 47.77±0.98% whereas CL9 liposomes, which were prepared with DSPC alone could release only 39.81±0.27%. Among the four-composition used in the present study using DSPC, liposomes prepared with DSPC without cholesterol could release the highest amount of drug followed by DSPC/cholesterol groups of CL10, CL11 and CL12 in a decreasing order. DSPC liposomes could retain 60.19% of the initial drug content whereas the formulations CL10, CL11 and CL12 were found to retain 64.51%, 68.89% and 77.19% of their drug content respectively. When the percentage of drug retained in MLVs were normalized to lipid content of each sample, CL9 liposomes (DSPC alone) was noted to retain the drug remarkably lower, whereas other liposomes could retain the drug at higher percentage, supporting the outcome that highest extent of drug release occurred in cholesterol free formulation. This trend of drug release may be due to the fact that cholesterol might have located itself to the glycerol backbone of the membrane pushing CLB to the inner core; which explains the decreased release rates with increasing concentrations of cholesterol [17]. The drug release was slowly progressing and not of the sudden burst in any of the prepared formulation.

When compared to the release profile of DSPC liposomes, the stealth liposomes have prolonged further the drug release with greater %EE (**Table 1**). This slower release in case of stealth liposomes could be corroborated to the facilitated hydration process owing to PEG presence on the surface of the vesicles.

#### 3.1.10. Release kinetics

The release kinetics of CLB from various liposomes was determined by applying mathematical equations such as zero order, first order, Higuchi’s square root of time and Korsmeyer-Peppas model which were used by linearization of dissolution profile and comparing their correlation coefficients respectively. The first two mathematical equations were applied in order to find out the order of release. The plot of %cumulative drug release *vs* time was found to be linear with greater correlation coefficient (r^2^ = 0.98–0.998) than that of first order kinetics (r^2^ = 0.557–0.873) indicating that drug release is independent of concentration and liposome formulations release the same amount of drug by unit of time. Then, to gain further insight into CLB release mechanism from liposomes, the release profile was fitted to Higuchi’s and Korsmeyer-Peppas models and analysed. The release data was found to fit well with Higuchi’s model with better linearity and correlation coefficient of 0.959–0.990 than that of first order kinetics and correspond to zero order kinetics equation. This showed that CLB release mechanism from liposomes was diffusion controlled. Further Korsmeyer-Peppas equation showed good correlation with experimental data. The Peppas model is used generally to locate the release mechanism precisely. The release exponent ‘*n*’ is used to predict the diffusion release mechanism. If the release exponent *n* = 0.5, the drug transport mechanism is by Fickian diffusion, and for higher values of *n* between 0.5 and 1.0 (0.5 < *n* < 1.0), or *n* = 1.0 or *n* > 1.0, it is considered as non-Fickian model and the drug transport mechanism is by anomalous or Case-II (purely relaxation-controlled delivery) or Super Case-II transport, respectively [26]. This approach has also been implied for liposomes with PE-PEG layers [36].

Thus, non-Fickian type of diffusion is considered as the main mechanism of CLB release from liposomes. This may be due to interfacial structure of PEGylated liposomes (i.e., tethered PEG chains at the lipid head group surface) plays an additional role in drug transport process. Non-Fickian type of diffusion is otherwise known as anomalous diffusion; which is the coupling of diffusion, interfacial barrier action and erosion, and indicates that drug release is controlled by more than one of aforementioned process.

### 3.2. *In vivo* performance of CLB liposomes

The best liposomes of each category such as conventional liposomes, liposomes prepared with long alkyl chain lipid DSPC and stealth liposome formulations were designated as CL, DSPCL and SL respectively to describe the *in vivo* studies conveniently. *In vivo* performance of liposomes was verified by carrying out (i) assessment of anti-inflammatory activity, (ii) assessment of analgesic activity, (iii) pharmacokinetic study and (iv) bio-distribution study.

#### 3.2.1. Assessment of anti-inflammatory activity

**Fig 8:**
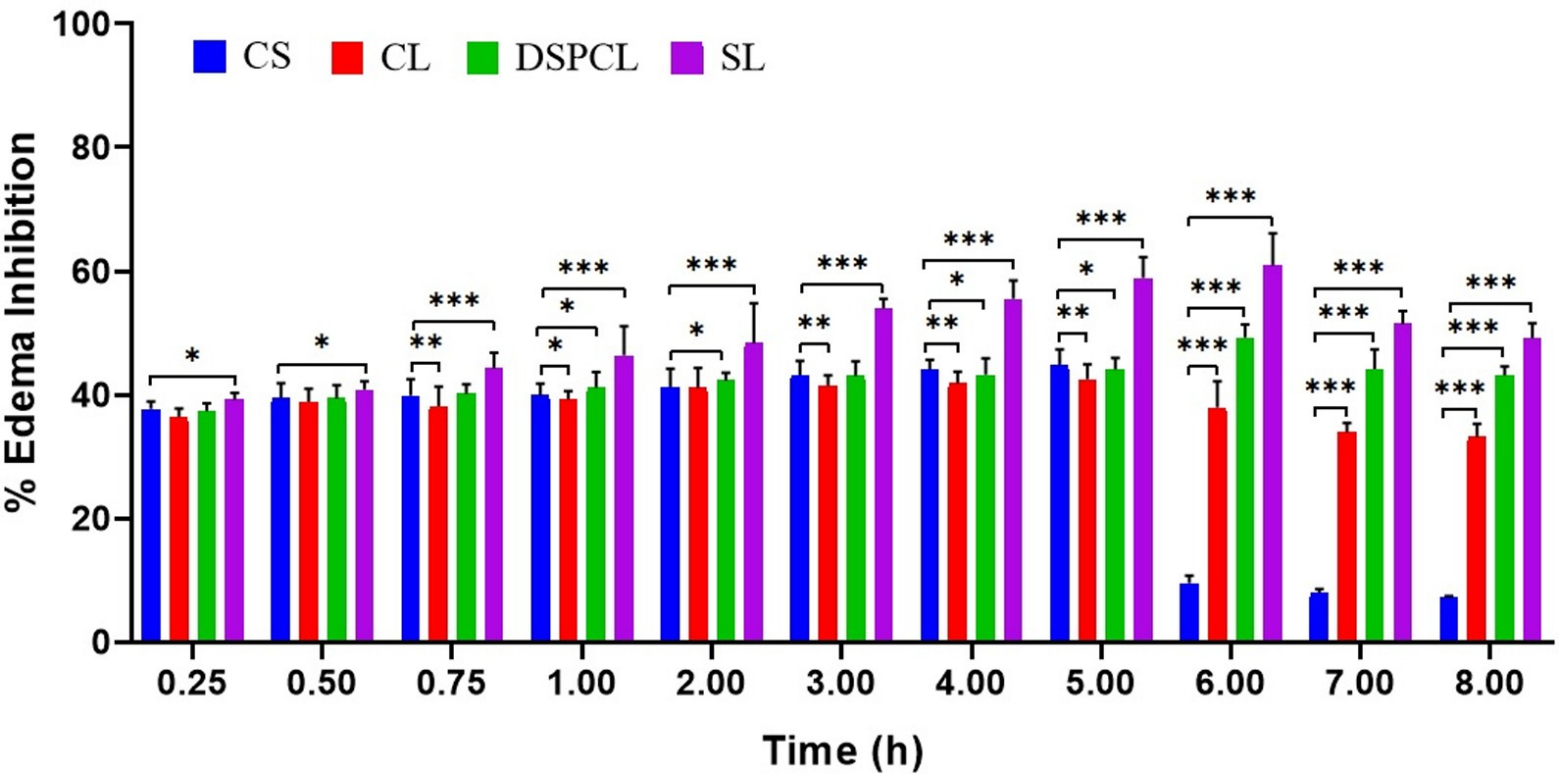
Inhibition percentage of CLB conventional liposome (CL), CLB DSPC liposomes (DSPCL) and CLB stealth liposomes (SL) compared to free CLB solution (CS) (data expressed as mean ± SD, *n* = 6; *p<0.05, **p<0.01, ***p<0.001).

Carrageenan induced rat paw edema test was used to examine the *in vivo* effects of formulations under study. Intra plantar injection of carrageenan caused a time dependent paw edema in rat. The CLB loaded stealth liposomes (SL; the best stealth liposome formulation, CL13) not only decreased the inflammation to the larger magnitude, but also sustained this magnitude (**Fig 8**). This formulation maintained the % inhibition above 50% from 3–7 h and 49.12% at the end of even 8 h. The maximum % inhibition was observed at 6 h i.e. 61%. The formulation DSPC liposome (DSPCL; the best liposomes prepared with long alkyl chain lipid DSPC, CL10) has shown above 40% of inhibition from 1–8 h. The maximum inhibition was observed at 6 h i.e. 49%. Whereas, the conventional liposome formulation (CL; the best conventional liposome, CL5) has shown lower magnitude of action compared to other two liposomes. However, in case of free drug solution maximum inhibition was displayed at 5 h with the magnitude of 44.82% and just after 5 h it scored below 10%. The possible reason could be drug concentration in blood which was maintained for longer duration in case of stealth as well as other liposome formulations in comparison to the plain drug. But maximum inhibitory effect was observed in case of stealth liposomes. This may be due to the fact that less or no aggregation or fusion of stealth liposomes owing to PE-PEG coating which might have increased the circulation time of liposomes in blood and would have caused passive targeting into inflammatory area by extra vascularization through the gaps formed between the endothelial cells of vasculature.

The data is expressed as mean ± SD (*n* = 6) and statistically assessed by one way analysis of variance (ANOVA). Values for edema rate percentage for liposomes were compared to the saline control and the differences were determined statistically using Dunnett’s *t* test. *p* < 0.05 was considered as statistically significant.

#### 3.2.2. Assessment of analgesic activity

**Fig 9:**
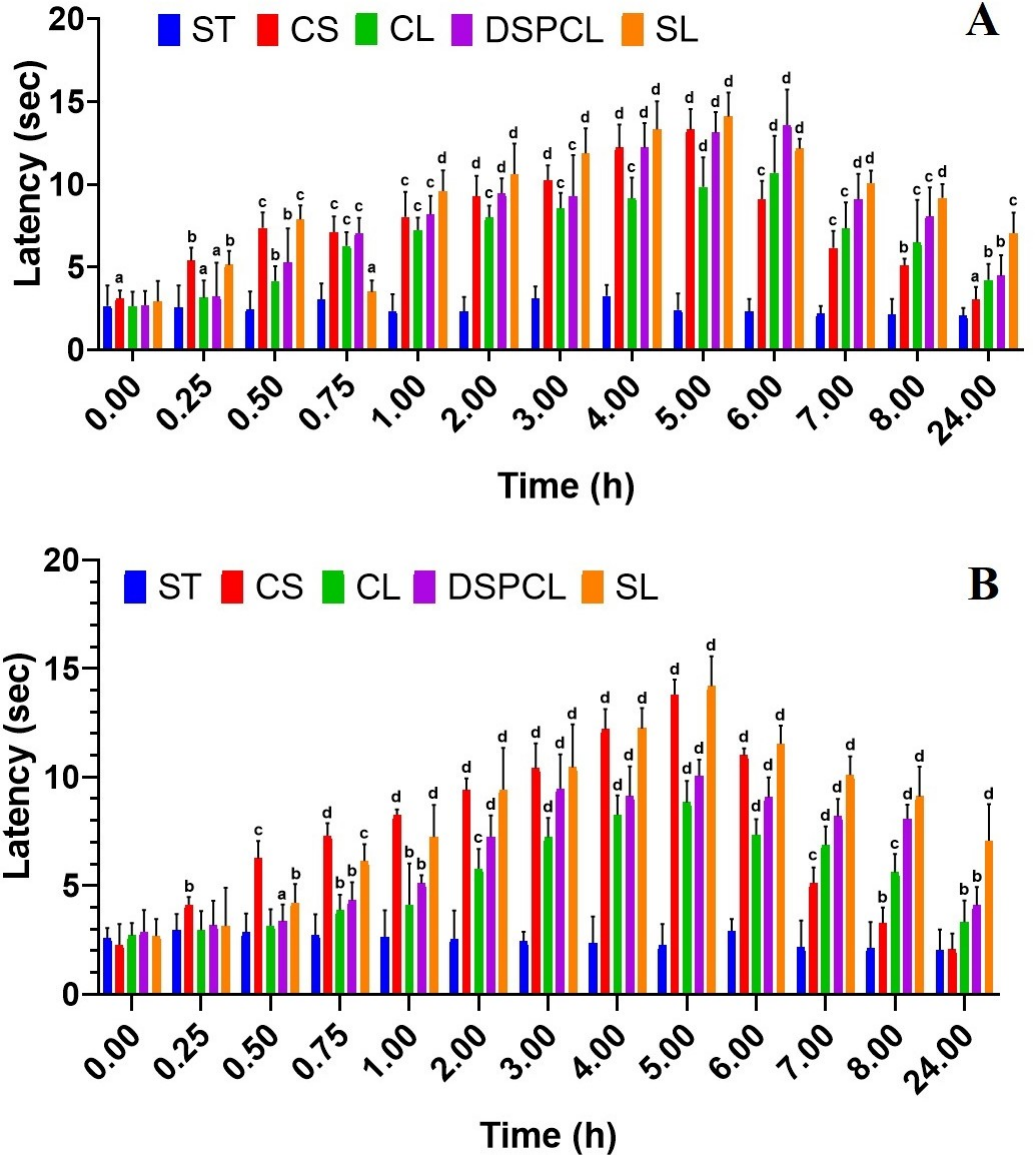
Analgesic activity outcomes of free CLB solution (CS), CLB conventional liposome (CL), CLB DSPC liposomes (DSPCL) and CLB stealth liposomes (SL) compared to saline treated control (ST) using (A) tail flick test and (B) hot plate test (Data expressed as mean ± SD, *n* = 6; a-*p*<0.05, b-*p*<0.01, c-*p*<0.001, d-*p*<0.0001).

*3*.*2*.*2*.*1*. *Tail flick test and hot plate test*. All the liposome formulations have increased the tail flick and hot plate latency when compared to the control groups. The latent period of control group animals did not change much in both tail flick test and hot plate test (**Fig 9A** and **9B**). In the hot plate test and tail flick test, maximum analgesic response was observed at 5 h, i.e. 14 sec and the response of 7 sec was maintained at the end of even 24 h for the stealth liposomes. For DSPCL and CL, the analgesic response was found to be lesser than stealth liposomes. However, DSPCL and CL formulations were also noted to maintain 4 sec latent time even at 24 h. On the other hand, the plain drug solution has shown the maximum effect at 5 h i.e. 13 sec, after which the response decreased drastically and lost completely at the end of 24 h.

#### 3.2.3. Pharmacokinetic study

**Fig 10:**
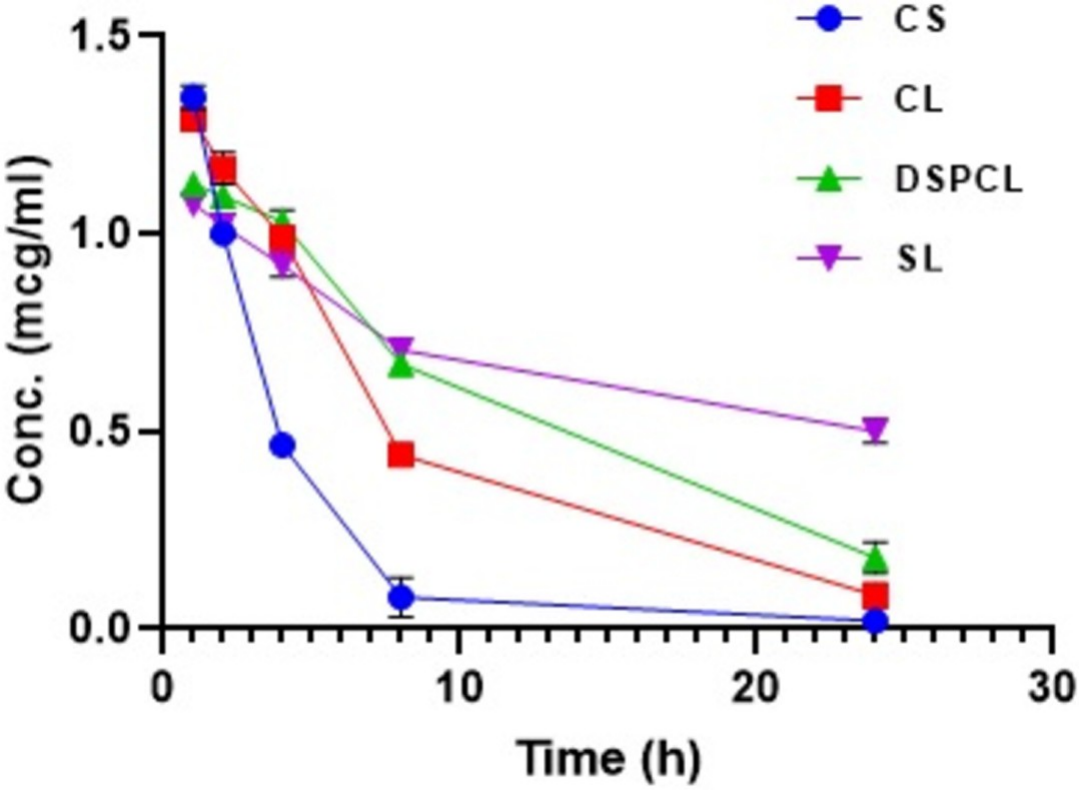
Plasma concentration-time profile of various CLB liposomes and free drug solution (data expressed as mean ± SD, *n* = 3).

There was a marked increase in area under CLB concentration *vs* time curve of stealth liposomes i.e. 16.42±0.57 μg/mL/h than the other liposomes such as CL and DSPCL. The AUC of free CLB solution (CS) was significantly lower than all the liposome formulations i.e. 5.20±0.8 μg/mL/h. The results indicated that there was slow and steady clearance of CLB in case of liposomes and with regards to the stealth liposomes the clearance was further less comparatively. The clearance was 190.48±32.27 mL/h/kg for free drug solution (CS) and it was 85.08±8.47 mL/h/kg, 62.31±6.49 mL/h/kg for CL and DSPCL formulations respectively. The difference in clearance was found to be significant for stealth liposomes (30.66±3.17 mL/h/kg) compared to the other liposomes. Both conventional liposomes (CL) and liposomes with long alkyl chain lipid (DSPCL) showed increased MRT and elimination half-life when compared to CLB solution (CS) but for long circulating stealth liposomes (SL) both the aforementioned parameters were further increased considerably (**Table 5** and **Fig 10**). Thus, increase in t_1/2_, MRT, AUC and decreased rate of clearance observed for stealth liposomes implies the extended bioavailability and consequent efficacy.

**Table 5:** Pharmacokinetic parameters of free CLB solution (CS), conventional liposomes (CL), DSPC liposomes (DSPCL) and stealth liposomes (SL) in arthritic rats (dose 1 mg/kg).

Parameter	CS	CL	DSPCL	SL
AUC_0-t_ (μg/mL/h)	5.20±0.8	11.36±0.53	14.01±0.97	16.42±0.57
Elimination half-life (t_1/2_) (h)	4.16±0.64	5.83±0.66	8.15±0.78	22.62±2.63
Clearance (mL/h/kg)	190.48±32.27	85.08±8.47	62.31±6.49	30.66±3.17
MRT (h)	4.42±1.07	7.72±1.03	11.20±1.30	33.37±3.59

Data expressed as mean ± SD, *n* = 3

#### 3.2.4. Bio distribution study

Bio-distribution study was conducted for 24 h for SL and other formulations, comparing with the free drug solution. In liver, conventional liposomes (CL) and DSPC liposomes (DSPCL) were distributed mainly within 1 h of administration. This may be due to the fast recognition of the liposomes towards reticuloendothelial system (RES). Percentage of injected drug deposited in liver was increased up to 8 h and maximum was 47% and 42% respectively for CL and DSPCL. In case of SL, the drug localization in liver was very much minimum when compared to other liposomes and free drug solution. Only 18.89% of injected dose was found to be localized in liver at 8 h for stealth liposomes, and for free drug solution (CS) it was noted to be 28.58% (**Table 6**). Considerable amount of drug was also deposited in spleen at 8 h for all the liposomal formulations. 21.67% of injected dose for CL and 17.34% of injected dose for DSPCL were noted to be residing in the spleen. It scored only 1.9% in case of stealth liposomes. 5.5% and 3.9% of injected dose respectively were mounted in kidney at 8 h for CL and DSPCL. This was only 0.71% in case of stealth liposomes at t_max_ of 8 h. In contrast to this, in case of free drug solution C_max_ was achieved within the 1 h.

**Table 6:** Recovery of CLB as % of administered dose in different organs at various time intervals following i.v. injection of free CLB solution (CS) and CLB stealth liposomes (SL) to arthritic rats (dose 1mg/kg).

% Recovery from Organ	Time (h)				
	1	2	4	8	24
CS					
Liver	5.49± 0.52	10.35±0.41	19.22±0.14	28.58±0.40	3.435±0.45
Spleen	0.175±0.08	0.145±0.05	0.14±0.07	0.085±0.02	0.08±0.03
Kidney	2.445± 0.26	2.13±0.14	1.755±0.16	1.395±0.23	1.05±0.1
Paw	0.0095±0.01	0.018±0.007	0.012±0.002	0.0052±0.003	0.002±0.01
SL					
Liver	5.375±0.21	10.595±0.35	13.785±0.33	18.885±1.05	3.565±0.22
Spleen	1.195±0.05	1.905±0.02	2.435±0.12	1.905±0.08	0.935±0.04
Kidney	0.48±0.08	0.6±0.03	0.68±0.04	0.715±0.05	0.31±0.03
Paw	1.975±0.013	1.835±0.058	1.626±0.032	1.264±0.109	0.415±0.013

Data expressed as mean ± SD, *n* = 3

Since the aim of entrapping CLB into stealth liposomes is to increase the therapeutic availability of the drug at the inflammatory sites of arthritic joints and to reduce the RES uptake, concentration of CLB in arthritic joint was found out up to 24 h. For all the formulations maximum drug levels were attained in paw after 1 h of administration. Only 0.0095% of drug was found in paw after 1 h of administration of free dug solution, whereas for CL and DSPCL, 0.052 and 0.094% of administered drug was detected. However, 1.975% of drug was localized in paw for stealth liposomes. This outcome could be attributed to PEG surface coating and related steric hindrance, preventing sufficiently the opsonization of liposomes with plasma components and leading to greater accumulation of drug into inflamed region by passive targeting [37].

Overall, conventional liposomes and DSPC liposomes were accumulated substantially in the liver and the spleen, although the size of liposomes was considerably less. These outcomes indicated that stealth liposomes could remain in blood for prolonged time with a reduced liposome uptake by RES, and lesser accumulation in kidney; thereby reducing the possibility of risk of toxicity to RES and renal organs and increase the accumulation of drug in inflammatory area.

## 4. Conclusions

CLB loaded stealth liposomes are a favourable system for minimizing systemic toxicity while maximizing the therapeutic effect of CLB. In the present study, several liposome-based formulations were prepared after optimizing component proportions, and process and formulation parameters. Amongst them, CLB loaded stealth liposomes (CL13) had shown greater % encapsulation efficiency of 94.2±0.8% with the vesicle size of 0.149±0.25μm and with good stability for 6 months. Intravenous administration of CLB loaded stealth liposomes would remain in circulation for a prolonged period and can passively target the sites of inflammation owing to improved permeability and retention effect. Pharmacodynamics, pharmacokinetics and biodistribution studies of stealth liposomes (CL13) further confirmed that the high amount of administered drug dose was accumulated at the inflammatory site with minimum exposure to other non-target organs and tissues.
